# Long-Range Dependent Traffic Classification with Convolutional Neural Networks Based on Hurst Exponent Analysis

**DOI:** 10.3390/e22101159

**Published:** 2020-10-15

**Authors:** Katarzyna Filus, Adam Domański, Joanna Domańska, Dariusz Marek, Jakub Szyguła

**Affiliations:** 1 Institute of Theoretical and Applied Informatics, Polish Academy of Sciences, Bałtycka 5, 44-100 Gliwice, Poland; kfilus@iitis.pl (K.F.); joanna@iitis.pl (J.D.); 2 Department of Distributed Systems and Informatic Devices, Faculty of Automatic Control, Electronics and Computer Science, Silesian University of Technology, Akademicka 16, 44-100 Gliwice, Poland; Adam.Domanski@polsl.pl (A.D.); Dariusz.Marek@polsl.pl (D.M.)

**Keywords:** neural networks, convolutional neural networks, Hurst exponent, self-similarity, Internet traffic, fractional Gaussian noise

## Abstract

The paper examines the ability of neural networks to classify Internet traffic data in terms of self-similarity expressed by the Hurst exponent. Fractional Gaussian noise is used for the generation of synthetic data for modeling the genuine ones. It is presented that the trained model is capable of classifying the synthetic data obtained from the Pareto distribution and the real traffic data. We present the results of training for different optimizers of the cost function and a different number of convolutional layers in the neural network.

## 1. Introduction

The Internet has certainly changed the world. During the era in which the information flow boosts the economic growth, safety and security have become the most important aspects of analysis and modeling of the Internet traffic. The traffic is the most important indicator of network load and status [1]. The time when the traffic was defined by the simple memoryless Poisson model has passed. In the early 1990s, it was first demonstrated by Willinger et al. [2] that the Ethernet Local Area Network (LAN) traffic is statistically self-similar. The Internet traffic at a packet level is in fact a superposition of numerous individual streams/connections between pairs of hosts. A fundamental physical cause of the self-similar behavior of the aggregated Internet traffic is a high variability of the ON and OFF phases of these individual streams/connections [3], which can be visualized by infinite-variance heavy-tailed distributions. ON–OFF behavior of a single host operation consists of ON (when packets are sent) and OFF (idle state) phases. Self-similarity and Long-Range Dependence (LRD) to this day are still relevant topics in the literature [4,5,6]. The analysis of these occurrences was recently applied to the attack detection domain [7,8,9]. The studies have proven that ignoring these phenomena has a negative effect on the estimation of performance measurements. This can result in mean queue length enlargement at buffers and packet loss probability increase [10]. Because of this negative impact on the network performance [4], these phenomena should be taken into consideration in the network management process.

The measure used to estimate the degree of self-similarity and LRD is the Hurst exponent (Hurst parameter), denoted by H. It was proven in [11] that an empirical relationship between Hurst exponent and the Packet Loss Rate exists. A study considering Voice over IP (VoIP) has shown that the increase in the Hurst parameter values resulted in the increase in the Packet Loss Rates and the degradation of the quality of voice. The authors proposed the possibility to treat the Hurst parameter as the Quality of Service (QoS) metric. Although there are many statistical methods for estimating the value of this parameter, some of them can be affected by irregularities in data [12]. The classical methods of estimating the Hurst parameter are usually computationally demanding, which makes it impossible to use them in online estimation in routers. For that reason, we propose the method using trained Artificial Neural Network models to accelerate the estimation process.

Artificial Neural Networks (ANNs) are a powerful tool that displays the ability to adapt to data, recognize patterns and ignore a potential inadequacy in a training dataset. The ANN is a computational method inspired by the biological neural networks that occur in the animal nervous system. Because of their versatility, ANNs have found applications in many different fields. These applications include computer vision, text and time-series analysis and data generation [13]. More complex ANN architectures are used in healthcare, industry and defence, and, in some cases, they can even outperform humans in the advanced planning tasks [14]. Deep neural networks, a subgroup of ANNs, have shown the capability of providing high accuracy in a great variety of classification problems.

**Figure 1:**
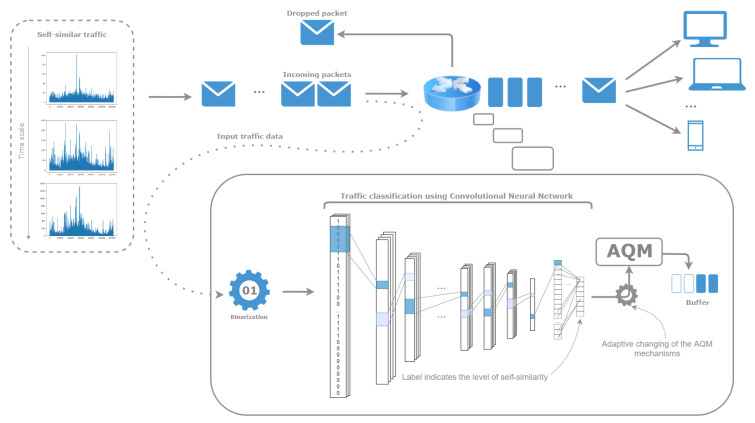
The scenario of using a machine learning model—the convolutional neural network—in a router to adaptively change the active queue management’s mechanisms in the event of self-similarity level fluctuations

In this paper, we present a solution utilizing the convolutional neural networks to classify the simulated network traffic based on an assessment of the degree of its self-similarity expressed by the Hurst exponent. Inspired by the adaptability of ANNs, we assume that they are capable of classifying data that have not been transformed in any computationally-demanding manner. To the best of our knowledge, the presented approach is a novel one to the analysis of network traffic. Ultimately, our goal is to create a model for forecasting traffic fluctuations and detecting anomalies based on past measurements. Such a model could be used as an indicator in the process of controlling the information flow in routers. The current Internet demands low latency and high performance for different types of traffic flow. Modern routers should detect changing network conditions and adapt to this environment. Taking into consideration a significant impact of the Hurst parameter on the QoS, the idea is to use information gained in the classification process to change the Active Queue Management (AQM) mechanisms adaptively. The model used in a router should be as fast as possible, considering the fact that it is used for real-time data. Thus, our goal is to create a network that is as computationally efficient as possible and accurate at the same time. Such an exploitation is presented in Figure 1.

To train the neural network model, it is necessary to supply it with a sufficient amount of data. The access to such an amount of real traffic data is limited; thus, it was necessary to provide synthetic data for modeling the real ones. An appropriate traffic source should display self-similarity and LRD. To model processes with LRD, the class of heavy-tailed distributions are usually used. An example of such a distribution is the Pareto distribution. It can be parameterized by a heaviness of the tail, which is associated with the Hurst parameter [15]. Fractional Gaussian Noise (FGN) is a stationary Gaussian process that is exactly self-similar. For that reason, it has become common to use FGN in the network traffic modeling [10]. An alternative is to use the Fractional Auto-regressive Integrated Moving Average (FARIMA). However, a disadvantage of this method is that it is computationally demanding [16].

Data used in the training process were generated from the FGN traffic source. To test the model, synthetic self-similar traffic was used (generated from the FGN-based and the Pareto-based sources). We also used the real traffic data collected at the Bellcore Morristown Research and Engineering Center (MRE) [2] for the analysis of the results. The second source of real traffic data contains the information about wide-area traffic between Digital Equipment Corporation and the rest of the world [17]. Both datasets can be found in the Internet Traffic Archive.

The remainder of the paper is organized as follows. Section 2 describes the current state of the art in this field. Section 3 is a brief introduction to the theory related to the issue: self-similarity, FGN, methods of estimating the value of the Hurst exponent and the neural networks. Section 4 is a description of the structure of the Neural Network model, the data and the experimental methods used to obtain the results for the purpose of this paper. In Section 5, there is a description of the results of the conducted experiments. Section 6 presents the conclusions drawn from the experiments and future work.

## 2. Related Works

The classical approach to LRD analysis involves using statistical methods to estimate the value of the Hurst exponent. There are many traditional methods of estimation, including R/S plot, aggregated variance, periodogram-based method, detrended fluctuation analysis, local Whittle’s estimator and wavelet-based method. These methods utilize different approaches in the process of estimation, and, as a result, the spectrum of the values can be wide. These statistical approaches are still used in the domain, e.g., R/S method [5,6,7], wavelet-method [8] and detrended fluctuation analysis [18]. In many studies, it has been proven that the wavelet-based technique gives the most reliable results [8,10]. Another well-established tool for the detection of LRD in both stationary and non-stationary time series is detrended fluctuation analysis [19].

In the majority of related works, the Hurst parameter estimation has only been used as an ancillary mechanism to assess the predictability of the time-series with ANNs [20,21,22,23]. Qian and Rasheed [20] used neural networks (with one hidden layer) to predict time series describing financial data. Hurst exponent (estimated with R/S method) was used to classify data into three categories: a random series, an anti-persistent and a persistent series. It was shown that the prediction of the time series is more accurate when the value of the Hurst parameter is closer to one. Rutka [21] proposed a traffic prediction model and based the assessment of the predictability on the aggregated variance method. The data were aggregated with a different level of window sizes. For the different levels, the performance of the models (multilayer perceptrons and radial basis function networks) was compared. Li et al. [22] also described the classification of time series data on the basis of the Hurst parameter analysis. They used R/S method to assess the degree of Self-Similarity and then used the Long Short-Term Memory Networks and Convolutional Neural Networks (LCNN) to classify time series. LCNN uses a multi-branch structure to transform the input data of each branch. They used the multi-scale transformation and the Gaussian noise transformation. The data were processed in the separate branches and then joined to return the collective result. In [23], the authors aimed to verify the existence of a relationship between long-term memory in fractal time series and the prediction error of financial asset returns obtained by the ANNs. The degree of self-similarity was estimated using the rescaled range analysis method. To measure the prediction error, the Root Mean Square Error (RMSE) produced by the neural network was used. In [24], the Hurst parameter-based fractal analysis is used to choose the input intervals that exhibit self-similarity for the neural network models to improve their performance.

As shown in the paragraph above, numerous works have been presented in which the Hurst parameter estimation is used as an ancillary mechanism for the neural networks, but there are only a few works that use the neural networks to estimate the Hurst parameter value itself. In [25], the ANNs were used to approximate the Hurst parameter values. Input features were obtained by creating a logarithmic histogram of the packet interarrival times. Ledesma-Orozco et al. [12] described the estimation of the Hurst exponent with the ANNs. They used the fact that the power spectral density of the given time series is dependent on the value of the Hurst parameter. The input data were transformed using a chain of the low-pass filters and the power calculators. The obtained values of the power spectral densities were used as the ANNs’ input. Both approaches are function approximation tasks.

Our contribution:Inspired by the ability of deep neural networks, and more specifically convolutional neural networks, to easily recognize the patterns and adapt to the unknown data, the aim of this article is to examine the ability to classify almost unprocessed data from the synthetic and real traffic on the basis of the Hurst exponent. In contrast to other neural networks-related works, where the data must be prepared using different computationally-demanding methods, we assume that no demanding data formatting is necessary. The input data are simply the binarized time series—without any further transformations that are computationally-intensive and time-consuming. Neural networks are very powerful tools, and, by using them, we expect to achieve a satisfactory accuracy without any complex transformations. Moreover, we try to limit the size of the network for a further decrease in the number of operations to create a fast model. Eventually, we want to incorporate this approach as an indicator of the traffic fluctuations and anomaly detector to be used to change the Active Queue Management (AQM) mechanisms adaptively. For that reason, the model should be as computationally efficient as possible, even if it means that we have to establish a reasonable trade-off between the simplicity of the model and its accuracy. In previous works, neural networks were used in a standard (for the Hurst parameter estimation) approach—as a continuous function approximation, which is a regression task. Contrary to this approach, we aggregate Hurst parameter target values in the intervals and the problem becomes a classification task. Discretized Hurst parameter values are more practical to indicate the necessity to change the AQM mechanisms because in this task we need the levels of self-similarity (intervals of the Hurst parameter) instead of the exact values. Additionally, to the best of our knowledge, it is the first time convolutional neural networks have been used in the Hurst parameter value estimation.

## 3. Preliminaries and Definitions/Background

To build a neural network model which accurately classifies the traffic data, it is important to understand the theory related to this issue. It consists of definitions of self-similarity and FGN, a presentation of the methods used to estimate the value of the Hurst exponent and a brief description of the neural networks theory.

### 3.1. Self-Similarity and Long-Range Dependence

The term “self-similarity” was first introduced in the 1960s by Mandelbrot. The object is self-similar if the portion of the whole object can—in a statistical sense—be considered a reduced-scale image of the whole. In other words, it means having the same statistical properties independently of the scale. This phenomenon can be frequently observed in nature. This occurrence is described as a case study of the scaled coastlines in Mandelbrot’s article.

Although phenomena of self-similarity and LRD are usually used interchangeably, they are not equivalent [10].

A continuous-time series Y(t) is exactly self-similar if it satisfies the condition:

(1) $$Y\left(t\right)\overset{d}{=}a^{-H}Y\left(at\right),$$

for t≥0,a≥0 and 0<H<1 [4]. This means that it is statistically invariable in different time scales. *H* is the Hurst exponent, which expresses the degree of self-similarity. A self-similar process displays LRD if it is an asymptotically second-order self-similar process. The occurrence of such a phenomenon entails the presence of temporal similarities in the time series [26]. Let $X_{n}$ be a second-order stationary process. LRD occurs when the autocorrelation function of this process ρ(k) follows a power law [26]:

(2) $$\rho \left(k\right)∼k^{-\beta }\;as\;k\to \infty ,$$

which indicates a slow decrease of the function as k→∞. The quantity ρ(k) can be computed as follows:

(3) $$\rho \left(k\right)\overset{\Delta }{=}\frac{r(k)}{r(0)},$$

where r(k) is a covariance function:

(4) $$r\left(k\right)=\mathrm{Cov}\left(X_{n},X_{n-k}\right)\overset{\Delta }{=}E\left[\left(X_{n}-\mu \right)\left(X_{n-k}-\mu \right)\right]$$

The process is self-similar and displays the LRD for the values of the Hurst exponent between 0.5 and 1.

### 3.2. Fractional Gaussian Noise

FGN is a Gaussian, exactly self-similar process frequently used in network performance evaluation [10]. Let $B_{H}\left(t\right)$ be the Fractional Brownian Motion (FBM) process. The increment process, $X\left(t\right)=B_{H}\left(t+1\right)-B_{H}\left(t\right)$, is called FGN.

The autocorrelation function of the FGN process is given by [10]:

(5) $$\rho^{\left(m\right)}\left(k\right)=\rho \left(k\right)=\frac{1}{2}\left[{\left(k+1\right)}^{2H}-2k^{2H}+{\left(k-1\right)}^{2H}\right],$$

This condition is sufficient for the second-order self-similarity.

The value of *H* determines the kind of the process under examination:

- H∈(0;0.5): the process is negatively correlated, which means the LRD does not occur.
- H=0.5—the process is uncorrelated.
- H∈(0.5;1)—the process is correlated, which means that the LRD occurs.

In this paper, to generate the data, the method from the Python library stochastic is used. The algorithm used to generate the FGN samples is the Davies–Harte algorithm.

### 3.3. Hurst Exponent Estimation

There are many methods of estimating the value of the Hurst exponent. This value can be utilized to classify the time series in terms of self-similarity and LRD. To estimate the values of the parameter, it is possible to use one of the methods below:

- Aggregate Variance Method;
- R/S Analysis;
- Periodogram-Based Method;
- Detrended Fluctuation Analysis;
- Local Whittle’s Estimator; and
- Wavelet-Based Method.

To estimate the values of the parameter to label the data, the method called detrended fluctuation analysis [27] is used in this paper. An advantage of this method is that it can ignore trends of a different order. It is important to use the method that can make distinctions between long-range dependencies and trends occurring in the examined data. The occurrence of the strong trend can be mistakenly identified as a long-range correlation [19].

**Detrended fluctuation analysis** is a statistical method utilized to determine the self-similarity of the series. The exponent given by this method is similar to the Hurst exponent, but it can also be applied to the non-stationary processes. Let $x_{t}$ be the time series of length N, where t∈N. The process $X_{t}$ is a cumulative sum of the series:

(6) $$X_{t}=\sum_{i=1}^{t}\left(x_{i}-m\right),$$

where *m* is the mean [27]. A time series of length N is divided into a number of shorter time series of length n samples each, and the local trend is calculated. The root-mean-square deviation—fluctuation—from the trend is calculated as follows [27]:

(7) $$F\left(n\right)=\sqrt{\frac{1}{N}\sum_{t=1}^{N}{\left(X_{t}-Y_{t}\right)}^{2}}$$

where $Y_{t}$ indicates the piece-wise sequence of the local trend fits.

The slope of the straight-line fit of the log-log plot of the F(n) versus n gives α, which for α∈(0;1) equals the value of the Hurst exponent. If α>1, it means that the examined process is non-stationary. For α=1, the process is a pink noise.

### 3.4. Deep Neural Networks

An Artificial Neural Network (ANN) is a computational method inspired by the biological structures. Neural networks are composed of units called *neurons* connected by directed links. The *activation* propagates from one neuron to another through this link. The activation also has a numeric *weight* associated with it, which determines the strength and sign of weight of the connection. ANNs are trained utilizing an optimization process that requires a loss (cost) function to calculate the error of the model. The accuracy of the model is strongly dependent on the chosen cost function and the optimizer.

Traditional machine learning models are based on shallow learning, e.g., random forests, Support Vector Machines (SVM) and k-nearest neighbors [28]. Neural networks can be used as shallow or deep learning models. Conventional, shallow architecture can be defined as a network using a single hidden layer. Operations happening in this layer map the input data into another feature space with nonlinear transformations. When multiple hidden layers are used, such a model can be named a deep learning model. In contrast to shallow learning, deep learning models can better extract the representations from the data and are suited for the high-dimensional learning that utilizes massive amounts of data [29]. This strong ability to find information and patterns encoded in input features entails capturing spatial and temporal correlations [30].

There are many different types of layers, which can be utilized to build a neural network model. The most popular ones are: dense layers, recurrent layers and convolutional layers. To process the time-series data, usually, recurrent layers are utilized. For data with LRD, Long-Short Term Memory (LSTM) layers can be used. LSTMs are frequently applied in language modeling and speech recognition. The alternative for LSTM layers is to use the Gated Recurrent Unit (GRU) layers. They use the same principle during the learning process, but they are less computationally expensive. However, they do not have as much representational power as LSTMs [13]. Convolutional layers consist of several feature maps, so that multiple features can be extracted at each area of data. This type of layers uses the mathematical operation named convolution that is applied to local features [31]. To reduce the resolution of the feature maps, pooling layers can be used. The last pooling layer is in fact a global pooling layer that efficiently extracts global context features [32]. The purpose of machine learning is to create a model that can successfully perform on the unknown data, and that is why it is very important to prevent the model from overfitting. For that purpose, dropout layers can be used. It is one of the most effective regularization techniques for neural networks. To enable the model to generalize, it is also a good practice to use the normalization techniques. Batch normalization can adaptively normalize the data during the training process [13].

In recent years, convolutional neural networks have been used in many diverse areas, e.g., DNA–Protein binding sites prediction [33], magnetic resonance image reconstruction [34], automatic road segmentation [32], facial verification [35], music generation [36], relation extraction (extracting the semantic relationship between the pairs from plain text) [37], database intrusion detection [38], arc detection in pantograph–catenary systems [39] and the time-series classification [40]. This versatility of the CNNs makes them a reasonable choice to use while building a solution to the considered problem.

**Figure 2:**
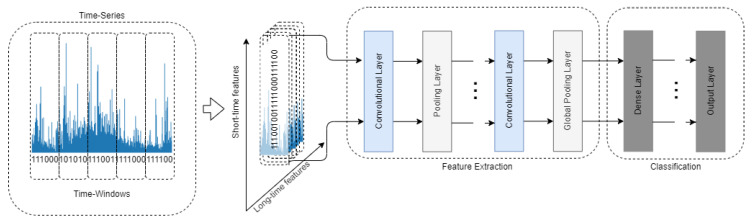
The conceptual structure of a convolutional neural network used for the purpose of time-series analysis

To process the time-series data with the convolutional layers, time is treated as a spatial dimension. One-dimensional convolutional layers can be a fast alternative to recurrent neural networks for time-series classification tasks [13]. Training the network with data transformed in such a manner usually consists of two stages. Operations in convolutional and pooling layers are used in turn to generate deep features of the raw data. The second stage engages the connection of the convolutional part with the classic Multi-Layer Perceptron (MLP). The MLP part is responsible for classification [40]. To visualize this process, the concept of training the CNN using the time-series data is shown in Figure 2.

## 4. Proposed Method

To create a model classifying the synthetic and the real traffic data, we propose a neural network structure with the convolutional layers. For pooling, the average pooling has been used. After every convolutional layer, the dropout layer was placed to prevent the model from overfitting and enable it to make generalizations. To implement the models, components from Python library Keras were used.

The data used in the training were synthesized with a generator using the FGN process. Every sample is a series of binary values representing the packet arrivals of length 10,000. Rea et al. [41] showed that the mean squared error of the estimation for different methods decreases with a number of data points in a time-series; taking a sample with 10,000 is a sufficient condition to assume a stable value of the Hurst parameter. This length of a time-series was also used in a study evaluating different methods of estimation of the value of the Hurst parameter [42]. For the purpose of the analysis, we divided this time-series into time slots that allowed us to represented the arrivals in the binary form. If in a particular time slot a packet arrived, we set the corresponding value to 1 and 0 otherwise. The real traffic to be used as the neural network’s input should be a high time-resolution and a complete time-series (without losses) on the packet level. This format of data is suitable for the neural networks created. The training dataset consists of 43,200 samples, the validation dataset 5400 samples and the test dataset 5400 samples. The number of samples in particular classes was evenly distributed. The training data were labeled with estimates of Hurst exponent obtained using the DFA method.

We considered nine categories (classes) representing the intervals of the Hurst exponent values. The first class is devoted to low values of *H*, for which H∈〈0,0.53) (Class 0). The next seven classes include values from subsequent intervals of a 0.05 width (Classes 1–7). The last class contains the samples for which the Hurst exponent value is greater than 0.88 (Class 8). Two marginal classes represent wider ranges of the Hurst exponent due to the fact that the target of the examination is to determine the degree of LRD of the samples, which occurs for H∈(0.5,1〉. For that reason, the first class considers all the values below 0.5 and a short interval of values from the target interval. Samples with Hurst exponent values approaching 1 are hard to generate and rarely occur in real traffic data.

To test the model, we used data generated with a source using the Pareto distribution and the real traffic data: from Bellcore Morristown Research and Engineering Center (MRE) and Digital Equipment Corporation (DEC). Data obtained from the Pareto source contain 3600 examples, MRE data 96 examples and DEC data 214 examples. Every example consists of 10,000 binary values. The test data were formatted in the same manner as training data and labeled with estimates of Hurst exponent obtained using the DFA method.

Models with a different number of convolutional layers were used to compare their accuracy. It was decided to use architectures containing two, three and four layers. As time can be treated as a spatial dimension to analyze the time series with convolutional layers, different divisions of data were used to determine the impact of this operation on the model’s accuracy.

**Figure 3:**
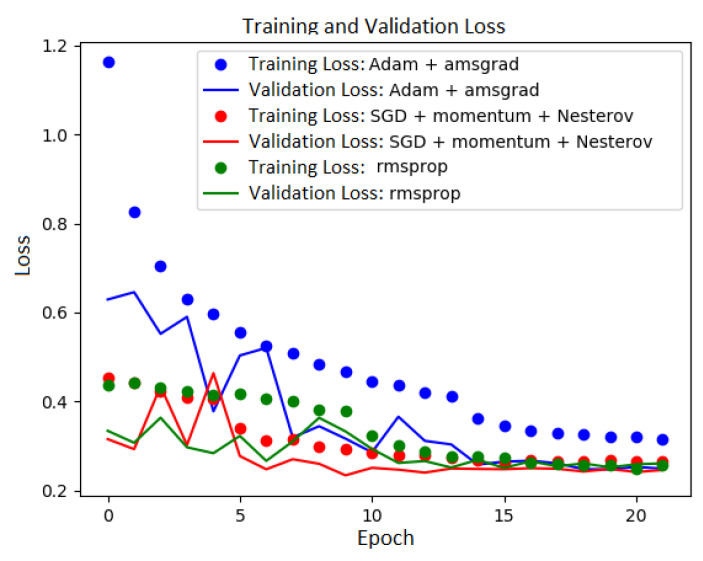
The training and validation loss during the training.

To train the models, three different optimizers were used to minimize the cost function: Adam optimizer, RMSprop and SGD (Stochastic Gradient Descent) with Momentum and NAG (Nesterov Accelerated Gradient). In Table 1, we present the settings used for the optimizers. We used one of the Keras Callbacks mechanism—ReduceLROnPlateau—to optimize the learning process. It reduces the learning rate by a factor 0.1 when there is no improvement in the validation loss with the patience of three epochs. It is one of the strategies to escape from the local minimum [13]. The cost (loss) function for every experiment was categorical cross-entropy, and it is given by the equation $Loss=-\sum_{i=1}^{N}y_{i}log{\hat{y}}_{i}$, where *N* is the length of the output values vector, $y_{i}$ is a target values vector, ${\hat{y}}_{i}$ is a predicted values vector and *i* denotes the *i*th position of the vectors. Models were trained on 22 epochs. To establish this value, we observed the behavior of the loss function during the training process. Figure 3 shows that the value of the loss function for all the plots stabilizes around the 20th epoch.

The results of the experiment contain the averaged accuracy values from the ten trained models. The given accuracy values represent the test accuracy for the trained model.

For the best-trained model in each group, we tested the accuracy using the data from the other sources: data generated using the Pareto distribution and the real traffic data. We performed an additional evaluation of the fastest—two-layer model (and the most accurate one in this group)—on two datasets with equal cardinality (3600 samples in total, 400 in each class) generated from the artificial traffic sources—the first one based on FGN and the second one—the Pareto distribution. For this analysis, we used confusion matrices and Receiver Operating Characteristics (ROC). ROC shows how true positive rates as a function of false positive rate changes for the different threshold values (it shows the classifier’s classification power). The confusion matrix allows us to more thoroughly examine the classification power for different classes (intervals of the Hurst parameter values). Although the examined task is a classification task, Hurst parameter values are continuous. Edge values in the intervals can be classified to neighboring classes and can decrease the model’s performance in terms of the accuracy and standard machine learning classification metrics. For that reason, the most valuable evaluation is to use confusion matrices and check the trend of the distribution of the most frequent outcomes of the classification (predicted values) in the confusion matrix.

## 5. Experimental Results

The results of averaged and highest testing accuracies on FGN data are included in Table 2 and Table 3. To treat time as a spatial dimension, it was necessary to transform one-dimensional arrays to two-dimensional ones. Table 2 shows the accuracy measure for the data transformed to dimension 50 × 200 and Table 3 for 1000 × 10.

The results of testing accuracies on data generated from the Pareto distribution source and real traffic data are included in Table 4 and Table 5. Table 4 shows the accuracy measures for the data transformed to dimension 50 × 200 to treat it as a spatial dimension and Table 5 for 1000 × 10.

The models achieve the highest accuracies when tested on data from the FGN source—the minimum accuracy for all models tested was 83.31%, and the maximum 96.94%. It is natural for the models to achieve the best results on such a dataset because they were trained on the data with the same origin. For data from a different artificial source (using the Pareto distribution). the minimum accuracy was 86.12% and the maximum 89.65%. For the real traffic data from Bellcore MRE. The minimum accuracy was 83.04% and the maximum 86.90%. Inferior results were obtained on the dataset from Digital Equipment Corporation: the minimum was 79.23% and the maximum was 83.28%. Although these results were below the others, they can still be perceived as satisfactory, considering the fact that the examined data describe real traffic and the models were trained on data from an artificial source.

All optimizers used to minimize the cost function performed well. The best results in terms of accuracy for data generated from the FGN source were achieved using the RMSprop optimizer: nine out of 12 averaged and highest accuracy measurements. The highest accuracy measurement using Adam optimizer was reached only once. On the other hand, the best accuracy measurements for the test data from sources other than the FGN one were reached using the SGD optimizer with Momentum and NAG (9 out of 18). The remaining optimizers performed equally well for such data.

We measured the execution time of the Hurst parameter estimation for 96 samples from Bellcore MRE using the detrended fluctuation analysis method and it was 15.25 s. The execution times for the convolutional neural networks were 0.12 s for a network with two layers, 0.16 s with three layers and 0.19 s with four layers (for the dimension 50 × 200 of data to treat time as a spatial dimension) and 0.19 for two layers, 0.21 for three layers and 0.26 for four layers (for dimension 1000 × 10). It is clearly visible that the speed of the neural network prediction is for the worst case almost 60 times and for the best almost 130 times faster than that achieved by using the statistical method.

**Figure 4:**
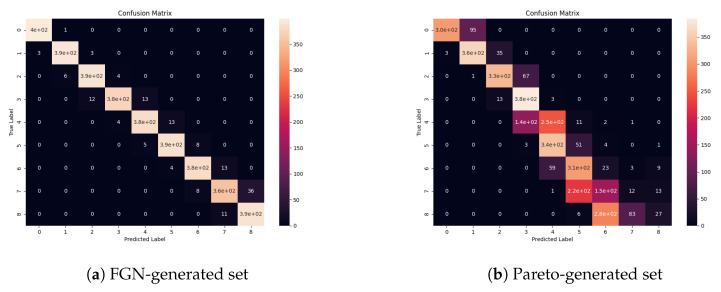
Confusion matrices of the most accurate two-layer model on the testing data generated from the FGN source and the source based on the Pareto distribution generated with the seaborn library.

**Figure 5:**
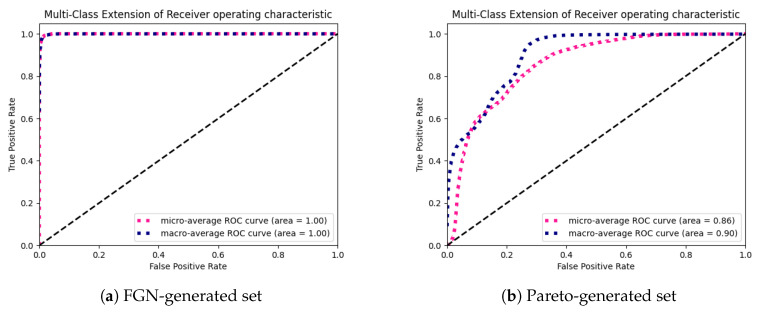
Receiver Operating Characteristic (ROC) of the micro and macro average results of the most accurate two-layer model on the testing data generated from the FGN source and the source based on the Pareto distribution.

We generated the confusion matrices (Figure 4) and ROCs (Figure 5) for two generated datasets (the first one using the FGN-based source and the second one using the Pareto-based source). The results show that the model exhibits almost a perfect classification power (Figure 5a) on the FGN-generated dataset. The precision of the model tested on the FGN-generated data can also be observed in Figure 4a. Although the model performance on the Pareto-generated dataset (Figure 4b and Figure 5b) is lower in terms of categorical values classification, we can observe that the trend of the distribution of the most frequent predictions exhibits a proper shape and the predicted values for particular true values are mostly distributed in two neighboring classes. The most problematic samples are those with high values of the Hurst parameter. It can be caused by a different type of the generated data and the fact that it is hard to obtain the samples with high values of the Hurst parameter. On the other hand, it is clearly visible that, for lower values of the Hurst parameter, the estimated values tend to be overstated, and for the higher values understated.

All of the results above were conducted using Keras (version 2.3.0) with the TensorFlow backend (version 2.0.0). We ran the experiments on AMD Ryzen 72700X Eight-Core Processor (3.7 GHz).

## 6. Conclusions

In the paper, we describe a method using convolutional neural networks to classify time series data with Long-Range Dependence. This paper summarizes the theoretical work on the statistical methods of estimating the Hurst parameter and a novel approach to use the neural networks in the process of estimation.

We consider neural networks with different numbers of convolutional layers. Additionally, for every neural network architecture, we tested various optimizers of the cost function given by the Keras library implementation. The results achieved by individual optimizers are comparable; therefore, using different types of optimizers to train the models is a good practice to find the model with the highest accuracy score.

The aim of the article is to determine if neural networks are capable of classifying synthetic and real-world traffic data with minimal interference in the input data. The experiments showed that even simple convolutional neural networks with only two convolutional layers can successfully classify such data. There is no need to transform the data in any computationally demanding manner before passing them to the neural network—binarizing the input is a sufficient method.

Numerical testing results on artificial data from a different source than the one used in the learning process and real traffic data show that the proposed classifiers can achieve high accuracies and the speed exceeding that of classical methods. The testing accuracy score on data from the FGN source remained in the range between 83.31% and 96.94%, and for data from other sources (artificial and real ones) between 79.23% and 89.65%. The speed achieved by the fastest model was approximately 100 times faster than the one achieved by estimation with the detrended fluctuation analysis method. Although the results shown in the form of confusion matrices show that the classification power is lower for the traffic from a different source than the one used to obtain the training data, it is still a good indicator of the Hurst parameter value, because the predicted values are situated in the true class itself and the neighboring ones. For the lower values of the Hurst parameter, values tend to be overstated, and for the higher values understated. A similar behavior was observed by Grab and Petersons [43], who examined the utilization of the wavelet transform for the Hurst parameter estimation.

In future work, we plan to create a forecasting tool for network traffic based on the history of measurements. The model based on the work presented in this paper could be utilized to probe real-time traffic data, since the prediction time is much faster than the one needed to estimate the value of the Hurst parameter using the statistical methods. A representative amount of real traffic data should be used to validate the model, particularly with an evenly distributed number of samples between the classes. To improve the performance of such a model, transfer learning could be used. This approach is based on using a pre-trained model (e.g., on the FGN-generated data) and an additional training on a smaller dataset. Such an approach allows us to use a smaller amount of real traffic data to tune the model. Internet traffic is very heterogenous and all of its types cannot be generalized and treated in the same way. If we wanted to use this model in practise, we should adjust it (via transfer learning) using the most appropriate (for our case) real traffic data. Internet traffic forecasting is a crucial element in the process of providing accurate traffic flow management in the modern dynamically evolving networks. Using the information about the degree of self-similarity of the traffic should improve the efficiency of existing methods. Creating the tool that adaptively controls the heterogeneous traffic flow should meet the demands of the rapidly evolving Internet.

## Figures and Tables

**Table 1:** The values of the parameters used for optimizers used in the experiment. The following symbols are used: η is the learning rate, β is the momentum coefficient, $\beta_{1}$ is the exponential decay rate for the first moment estimates, $\beta_{2}$ is the exponential decay rate for the second moment estimates and ρ is the history and future gradient discounting factor.

Optimizer	Parameters
Adam	$\eta =10^{-3},\;\beta_{1}=0.9,\;\beta_{2}=0.999$
RMSprop	$\eta =10^{-3},\;\rho =0.9$
SGD + Momentum + NAG	$\eta =10^{-2},\;\beta =0.9$

**Table 2:** The averaged and highest accuracy measurements in percent for different optimizers for the models with two, three and four convolutional layers and the dimension 50 × 200 of data to treat time as a spatial dimension on test dataset generated from FGN source.

		Optimizer		
		**Adam**	**RMSprop**	**SGD + Momentum + NAG**
**Average**	2 layers	89.23	89.52	88.11
	3 layers	90.41	90.51	90.70
	4 layers	89.79	91.38	90.63
**Highest**	2 layers	90.41	91.61	89.02
	3 layers	91.17	91.30	91.04
	4 layers	91.54	92.11	91.54

**Table 3:** The averaged accuracy measurements in percent for different optimizers for the models with two, three and four convolutional layers and the dimension 1000 × 10 of data to treat time as a spatial dimension on test dataset generated from FGN source.

		Optimizer		
		**Adam**	**RMSprop**	**SGD + Momentum + NAG**
**Average**	2 layers	94.55	94.30	94.6
	3 layers	95.34	95.73	94.93
	4 layers	95.67	96.29	95.46
**Highest**	2 layers	95.11	95.02	95.11
	3 layers	95.70	96.11	95.57
	4 layers	96.69	96.94	95.91

**Table 4:** The accuracy measurements for test data from different sources for the models with two, three and four convolutional layers and the dimension 50 × 200 of data to treat time as a spatial dimension on test datasets generated from Pareto source and real traffic.

		Optimizer		
		**Adam**	**RMSprop**	**SGD + Momentum + NAG**
**Pareto**	2 layers	87.69	87.92	87.10
	3 layers	86.73	86.78	86.66
	4 layers	86.53	86.12	86.32
**Bellcore**	2 layers	83.04	83.39	83.74
	3 layers	83.63	83.86	84.09
	4 layers	84.33	84.33	83.86
**DEC**	2 layers	79.49	80.37	79.44
	3 layers	79.80	79.54	79.23
	4 layers	79.54	79.60	79.49

**Table 5:** The accuracy measurements for test data from different sources for the models with two, three and four convolutional layers and the dimension 1000 × 10 of data to treat time as a spatial dimension on test datasets generated from Pareto source and real traffic.

		Optimizer		
		**Adam**	**RMSprop**	**SGD + Momentum + NAG**
**Pareto**	2 layers	88.54	88.12	88.90
	3 layers	88.36	88.58	89.65
	4 layers	88.94	88.35	88.94
**Bellcore**	2 layers	86.20	86.20	86.90
	3 layers	86.20	85.50	85.96
	4 layers	85.26	85.61	86.43
**DEC**	2 layers	82.61	83.07	83.07
	3 layers	83.28	82.14	81.36
	4 layers	80.58	81.72	82.19
